# Intensity-Product-Based Optical Sensing to Beat the Diffraction Limit in an Interferometer

**DOI:** 10.3390/s24155041

**Published:** 2024-08-04

**Authors:** Byoung S. Ham

**Affiliations:** 1 School of Electrical Engineering and Computer Science, Gwangju Institute of Science and Technology, Gwangju 61005, Republic of Korea; bham@gist.ac.kr; Tel.: +82-62-715-3502; 2 Qu-Lidar, Gwangju 61005, Republic of Korea

**Keywords:** optical sensing, projection measurement, many-wave interference, higher-order intensity product

## Abstract

The classically defined minimum uncertainty of the optical phase is known as the standard quantum limit or shot-noise limit (SNL), originating in the uncertainty principle of quantum mechanics. Based on the SNL, the phase sensitivity is inversely proportional to $\sqrt{K}$, where K is the number of interfering photons or statistically measured events. Thus, using a high-power laser is advantageous to enhance sensitivity due to the $\sqrt{K}$ gain in the signal-to-noise ratio. In a typical interferometer, however, the resolution remains in the diffraction limit of the K = 1 case unless the interfering photons are resolved as in quantum sensing. Here, a projection measurement method in quantum sensing is adapted for classical sensing to achieve an additional $\sqrt{K}$ gain in the resolution. To understand the projection measurements, several types of conventional interferometers based on N-wave interference are coherently analyzed as a classical reference and numerically compared with the proposed method. As a result, the Kth-order intensity product applied to the N-wave spectrometer exceeds the diffraction limit in classical sensing and the Heisenberg limit in quantum sensing, where the classical N-slit system inherently satisfies the Heisenberg limit of π/N in resolution.

## 1. Introduction

Optical sensing and metrology have been one of the most important research topics in modern science and technology for precision measurement [1,2,3,4,5,6,7,8,9,10,11,12,13]. In optical sensing, high-precision measurements have been pursued in physics [1,2,3], chemistry [4,5], biology [6,7], medicine [7], and even the semiconductor industry [8,9] and military services [10,11]. The classical phase sensitivity or resolution limit is known as the shot-noise limit (SNL), which originates in the uncertainty relation between photon number and phase [12,13,14,15]. In SNL, the phase sensitivity is proportional to $1/\sqrt{K}$, where K is the intensity order of interfering photons in an interferometer or statistically provided measurement events. Thus, to increase the signal-to-noise ratio (SNR), a higher-order K probe light must be used. However, the demonstration of SNL for K > 1 has not been reported yet in interferometer-based optical sensing and metrologies. Although many-wave interference in a Fabry–Perot interferometer (FPI) or grating-based spectrometer is a well-known technique for high-resolution spectroscopy [16], it is still limited to the K = 1 case of SNL with high-end optical and electronic systems.

To beat the SNL, quantum sensing has been developed for nonclassical phenomena [17,18,19,20,21,22,23,24,25,26,27,28]. In quantum sensing, super-resolution [20] and supersensitivity [21,22] have been studied using nonclassical lights such as maximally entangled photons of N00N states [12,14] and squeezed lights [15]. The photonic de Broglie wave (PBW) using N00N state is a good example of super-resolution, satisfying the Heisenberg limit (HL) [23,24,25] and overcoming SNL, where supersensitivity is an independent issue [20]. Super-resolution is for phase sensitivity to resolve a frequency difference better. However, N00N state-based PBWs suffer from inherently inefficient generation processes limited by nonlinear optics [20,21,22,23,24] and nonperfect fringe visibility for N > 2, where N is the total photon number of an entangled pair [20,25]. Unlike N00N states, squeezed states cannot be used for super-resolution, even though they have been well adopted for supersensitivity in gravitational wave detection below the SNL [15]. Supersensitivity is for the sensing ability to detect a signal below the SNL, where a 10 dB gain over the shot noise is normally achievable nowadays using squeezed lights. Due to the prerequisite of nonclassical light, however, quantum sensing is not compatible with classical sensors or metrologies. Most of all, N00N-based super-resolution is far behind classical sensors due to the limited order N and the working condition of extremely noisy environments [26,27], where the maximum entangled photon number N achieved by a PBW is just N = 18 [28].

**Figure 1:**
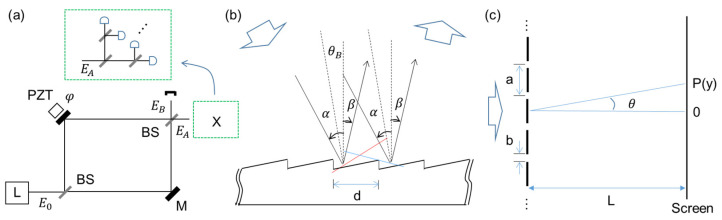
Schematic of various interferometers. (**a**) Intensity-product-based SNL. Inset X: projection measurements for the Kth intensity product. (**b**) Grating-based interferometer. (**c**) N-slit interferometer. L: laser, BS: beam splitter, PZT: piezoelectric transducer.

Here, the projection measurement in quantum sensing [25] is adapted to an interferometer for intensity-product-based optical sensing to show interferometric SNL beating the diffraction limit or Rayleigh criterion limited by K = 1. Satisfying coherence optics, the proposed method is inherently compatible with all interferometer-based sensors. The original projection measurement scheme is to split an interferometer’s output port into identical K ports using nonpolarizing 50/50 beam splitters (BSs), as shown in the inset of Figure 1a [29]. Here, the projection measurement aims to distinguish interfering photons in a post-detection manner, as originally understood in quantum sensing [17,25]. Thus, the role of divided fields in Figure 1a (see the inset for X) is to post-determine the number of interfering photons or measurement events contributing to the SNL on a single-shot measurement basis [29]. For the projection measurement, the maximum number K can be ideally equal to the photon number M of the input light L. Regarding the first-order (K = 1) intensity correlation of the Mach–Zehnder interferometer (MZI) output fields ($E_{A}$ and $E_{B}$), no difference exists between single photons and continuous-wave light [30], as demonstrated with a single photon for the same fringe [29,31]. This equality between quantum and classical approaches satisfies for K = 1 in Figure 1a without X [30]. Thus, the adaptability of the projection measurement to enhance the resolution overcoming diffraction limit is obvious due to K≫N, where the split number (or intensity product) K can be replaced by a 2D image sensor for block ‘X’ in Figure 1a.

## 2. Materials and Methods

Figure 1 shows the schematic of the proposed projection measurement-based optical sensing using higher-order intensity correlations (products) for the SNL. Figure 1a shows a typical MZI, and ‘X’ represents the projection measurement. For X, the MZI output is evenly divided into K ports by BSs for the Kth-order intensity correlation. Thus, ‘X’ is scalable by $2^{j}$, where *j* is the number of BSs. In the K-split MZI output ports, global phases generated by inserted BSs [32] and elongated optical paths to individual photodiodes do not affect their intensities due to Born’s rule, stating that measurement is the absolute square of its amplitude [33]. In other words, all K-divided output fields must be equal in intensity, satisfying the statistical ensemble of measurement events for the SNL on a single-shot measurement basis. Here, the statistical ensemble of K spit fields satisfies the Gaussian distribution of photon statistics.

## 3. Theory

For a coherence solution of the intensity-product-based optical sensing in Figure 1a, first, original MZI output intensities are derived as follows using the BS matrix [32]:

(1) $$I_{A}=\frac{I_{0}}{2}(1+cos\varphi ),$$

(2) $$I_{B}=\frac{I_{0}}{2}(1-cos\varphi ),$$

where $I_{A}=E_{A}E_{A}^{*}$, $I_{B}=E_{B}E_{B}^{*}$, and $E_{j}$ is the amplitude of the optical field. Thus, the MZI fringes show a deterministic coherence feature depending on the relative phase φ caused by the MZI path-length difference. Due to the same coherence feature, MZI in Figure 1 can be replaced by a Michelson interferometer, as usually adapted for remote sensors. Due to the global phase-independent intensities for all divided ports, the intensity of the jth divided output field in ‘X’ can be represented as:

(3) $$I_{j}={\left(\frac{1}{2}\right)}^{K}\frac{I_{0}}{2}(1+cos\varphi ).$$

**Figure 2:**
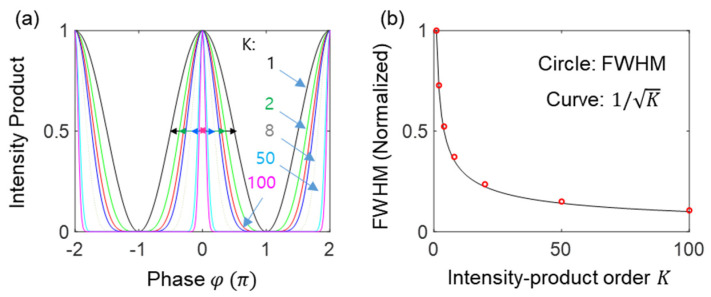
Numerical calculations of Equation (3) for K = 1 (black), 2 (green), 3 (red), 4 (blue), 8 (dotted), 50 (cyan), 100 (magenta). The ordered intensity products are normalized. (**b**) solid curve: 1/$\sqrt{K}$. Open red circles: data from the arrows (FWHMs) in the (**a**).

**Figure 3:**
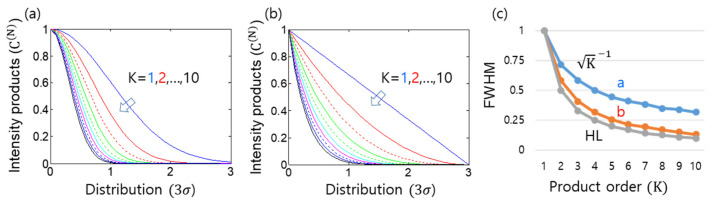
Numerical calculations of intensity-order-dependent FWHMs. (**a**) Gaussian. (**b**) Linear. (**c**) FWHM ratio vs. intensity order. a: for (**a**); b: for (**b**). HL: Heisenberg limit (1/N). $C^{\left(K\right)}={\left(I_{1}\right)}^{K}$.

Quantum mechanically, K implies interfering photons post-determined by the projection measurement: for a 1 W-power laser with 1 GHz bandwidth, the maximum photon number M is $10^{9}$. Using commercially available photodetectors whose response time is shorter than the inverse of the laser bandwidth, the Kth-order intensity correlation is as follows using Equation (3):

(4) $$C_{N}^{\left(K\right)}=\frac{I_{0}^{K}}{2^{(K+1)K}}{\left(1+cos\varphi \right)}^{K},$$

where K≤M. The numerical calculations of Equation (4) are shown in Figure 2, where the satisfaction of the output field by the Kth product is due to the Gaussian distribution of the measured light, as shown in Figure 3. Unlike quantum sensing using nonclassical light, the intensity product in Equation (4) can be coherently amplified, compensating for the reduction factor of $2^{-(K+1)K}$. Unlike the enhanced coherence effect in many-wave interference (discussed below), $I_{0}^{K}$ is the correlation effect by the intensity product [16]. This correlation effect is powerful in reducing unwanted noise. Satisfying K≪M in ‘X’ of Figure 1c, the intensity product in Equation (4) gives a great benefit to the resolution of the proposed optical sensing with a high SNR [29].

**Figure 4:**
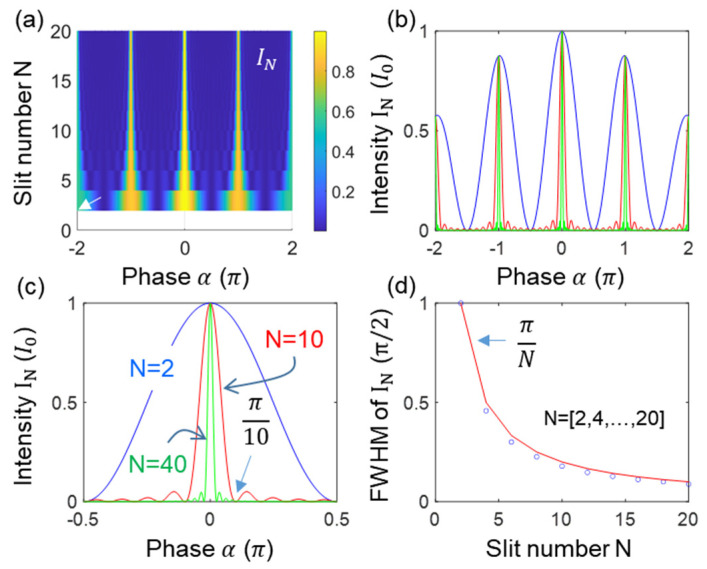
Numerical simulations of N-slit interference. (**a**) First-order intensity correlation $I_{N}(\varphi$). (**b**,**c**) Blue (N = 2), red (N = 10), green (N = 40). (**d**) Resolution (full width at half maxima). Red curve: π/N, a = 3b (see Figure 1c), and N = 2, 4, …, 20.

Figure 1b,c show schematics of many-wave interference on an N-groove grating and an N-slit interferometer, respectively. As introduced for MZI in Figure 1a, the interference fringe in Figure 1b,c can also be used for the same projection measurement, satisfying SNL. In Figure 1b,c, the N-groove or N-slit resulting interference fringes show an enhanced resolution by ${∆}_{N}=\pi /N$ (N≥2), where Figure 1a is only for N = 2 [16]. For this, a discrete phase relation between N coherent waves is essential [16]. Unlike the N-slit interferometer in Figure 1c, the N-groove grating in Figure 1b allows only one interference fringe in each grating order due to the nearly equal ratio of ‘a’ to ‘b.’ Thus, the well-known grating equation is given by $2dsin\theta_{B}=p\pi (\frac{p\lambda }{2})$ for the grating order and the ordered interference fringes, where p=0,±1,±2,…, and λ is the wavelength of the interfering light. For the N-wave interference, the analytical solution can be derived from the N-slit interferometer in Figure 1c [16]:

(5) $$I_{N}\left(\alpha ,\beta \right)={sinc}^{2}\beta {\left(\frac{sinN\alpha }{sin\alpha }\right)}^{2},$$

where $\alpha =k_{d}asin\theta /2$, $\beta =k_{d}bsin\theta /2$, and $k_{d}=2\pi /\lambda$. Slit number N must be fully covered by the coherent input light. As discussed below, Equation (5) results in N/2-enhanced resolution compared to the two-slit case of Equations (1) and (2) due to the N-wave superposition. Interestingly, this N-slit interferometer satisfies the Heisenberg limit for resolution [16]. The numerical calculations of Equation (5) are shown in Figure 4.

To overcome the phase resolution in Equation (5), satisfying the Heisenberg limit in phase resolution, the same projection measurement in Equation (4) is applied to K split output fields:

(6) $$R_{N}^{\left(K\right)}\left(\alpha ,\beta \right)=\frac{I_{N}^{K}}{2^{(K+1)K}}{sinc}^{2K}\beta {\left(\frac{sinN\alpha }{sin\alpha }\right)}^{2K}.$$

**Figure 5:**
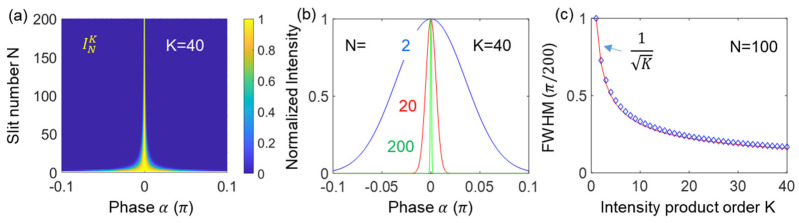
Numerical simulations of Kth intensity correlation in an N-slit interferometer. (**a**) N = 2~200, K = 40. (**b**) Normalized individual intensities for (**a**), where N = 2, 20, 200. K = 40. (**c**) Diamonds: K-dependent FWHM for N = 100 in (**a**). Red curve: SNL.

**Figure 6:**
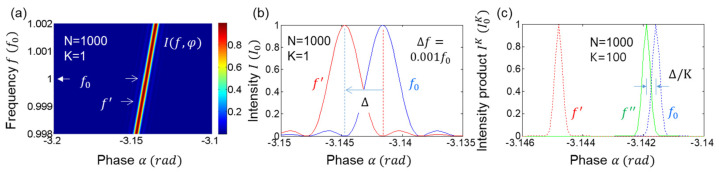
Numerical calculations of N-slit interference. (**a**) Intensity I(f,α) for N = 1000 and K = 1. (**b**) Details of (**a**) for frequency resolution. (**c**) Enhanced frequency resolution with intensity-product order K = 100.

Thus, the intensity product applied to the output field of Figure 1b or Figure 1c via the projection measurement method results in overcoming the resolution given by the Heisenberg limit. The corresponding numerical calculations are shown in Figure 5 and Figure 6.

## 4. Numerical Calculations

Figure 2 shows the numerical calculations of the Kth-order intensity correlations for Figure 1a using Equation (4). For this, the number of divided output ports is set at K = 100, where K is far smaller than the actual photon number M of $I_{0}$. All K-dependent intensity products are normalized for comparison purposes. As shown in Figure 2, the ratio of full-width-at-half-maxima (FWHM) of the Kth-order to the first–order (K = 1) intensity correlations is nearly $1/\sqrt{K}$ (see red circles in the right panel): the small discrepancy from the SNL theory (black curve) is due to the sine (monochromatic) function of the light rather than the Gaussian distribution of the actual laser light in photon statistics (discussed in Figure 3). Thus, the intensity-product-resulting resolution enhancement in Figure 2 demonstrates the SNL for Equation (4). In other words, Figure 2 verifies that the proposed projection measurement-based optical sensing in Figure 1a satisfies the SNL using a commercially available laser: for experimental demonstrations up to K = 4, see ref. [29]. More importantly, the resulting $\sqrt{K}$-enhanced resolution in Figure 2 does not require a photon-number resolving single-photon detector used in quantum sensing [12,13,14]. According to Born’s rule, the K-divided output field’s intensities in ‘X’ of Figure 1a are independent and individual for measurements, satisfying the statistical ensemble of the SNL. Due to the limited scalability of BSs (or 2D arrayed photodetector), far less than the actual photon number of the laser, the phase-resolution enhancement is even practical due to the high SNR, as in conventional sensors.

Figure 3a shows numerical calculations of FWHMs of the autocorrelation (self-intensity product) for a Gaussian function. Here, a laser has the same feature as the Gaussian distribution if the mean photon number is $\langle n\rangle \gg 1$. Figure 3b is for linearly distributed fields for a comparison purpose. The horizontal axis in Figure 3a,b is for the phase variation (noise) in the unit of standard deviation σ of the Gaussian function used for Figure 3a.

Figure 3c shows the ratio of the K-ordered FWHMs to the first order (K = 1) for Figure 3a,b, where ‘HL’ represents the Heisenberg limit in quantum sensing as a reference. Thus, the SNL is analytically confirmed for the Gaussian distribution of Figure 3a, where the FWHMs are inversely proportional to $\sqrt{K}$. This $\sqrt{K}$-enhanced phase sensitivity is due to the normal probability distribution of statistical events (see the blue dots in Figure 3c). Thus, the origin of the SNL in Figure 2 is the Gaussian distribution of a laser. If the probability distribution is linear as shown in Figure 3b, the resolution enhancement is much better than the Gaussian (see the red dots in Figure 3c). The enhancement factor in resolution can be higher if the photon distribution is non-Gaussian. Such an enhancement can be accomplished by frequency modulation as in a typical radar system [10,11]. Even in this case, the maximum sensing gain is still below the Heisenberg limit of quantum sensing, as shown by the gray curve in Figure 3c.

Figure 4 shows numerical calculations of Equation (5) for the many-wave interference in the N-slit system of Figure 1c. For the analysis, the slit number is set at 2≤N≤20. As shown in Figure 4a, the fringe condition is satisfied by α=pπ, where p=0,±1,±2,… As N increases, the fringe resolution improves. To understand the N effect, N-dependent interference fringes are shown in Figure 4b,c, where N = 2, 10, 40 are set for comparison purposes. From Figure 4a, FWHMs are calculated and plotted in Figure 4d. The red curve is the theoretical reference of π/N [16]. At a glance, both the numerical data from Figure 4a and the reference seem to match well. The small discrepancy, however, is due to the non-Gaussian function of $I_{N}\left(\alpha ,\beta \right)$ based on monochromatic waves, as discussed in Figure 3. Interestingly, this N-wave-caused resolution enhancement shows the same feature as the Heisenberg limit in quantum sensing [12,13,14,20,21,22,23,24,25].

Figure 5 shows numerical calculations of the Kth-order intensity products for Figure 1b,c and Figure 4. For this, Equation (6) is used, where two variables of N and K are set for 2≤N≤200 and 1≤K≤40, where N is far smaller than the actual photon number of $I_{0}$. All K-dependent intensity products $I_{N}^{K}$ are normalized for comparison purposes. As shown in Figure 5a–c, the ratio of FWHM of the Kth-order to the first-order intensity product is satisfied by $1/\sqrt{K}$ (see blue diamonds in Figure 5c), where the red curve refers to the SNL. The small discrepancy between them is due to the sine (monochromatic) function of the light rather than the Gaussian distribution of the actual laser light, as shown in Figure 2, Figure 3 and Figure 4. Thus, the intensity-product-resulting resolution enhancement in Figure 5c also demonstrates the same SNL applied to an N-slit interferometer. In other words, Figure 5 verifies that the proposed projection measurement-based optical sensing for the Kth-order intensity product is even effective for the fringes of a grating-based spectrometer. Due to the practically unlimited Kth order, the phase-resolution enhancement can beat the Heisenberg limit given by π/N (discussed in Figure 6) [12,13,14].

Figure 6 shows a practical example of the grating-based spectrometer with N = 1000. As discussed in Figure 4 and Figure 5, the resolution is enhanced by 500 times compared with the two-slit case. Figure 6a shows the interference fringe for Figure 1b or Figure 1c as a function of the frequency of the probe light and phase difference α (or position P on a screen focused by a lens). The frequency $f_{0}$ is a reference, where an unknown frequency detected by an arrayed photodiode is calculated with respect to the position, i.e., the phase difference α. As demonstrated in Figure 4 and Figure 5, the phase resolution by 1000 slits or grooves in Figure 6a is enhanced by π/1000, satisfying the Heisenberg limit in the amplitude version.

Figure 6b shows the resolving power to separate an unknown frequency $f^{\prime }$ from the reference $f_{0}$. The frequency difference of $f^{\prime }$ from $f_{0}$ is easily calculated by measuring the detuned phase Δ from the principle maxima at α=−π. Here, the frequency $f^{\prime }$ is chosen for $0.999f_{0}$ in Figure 6a. As shown in Figure 6b, $f^{\prime }$ is resolvable by the Rayleigh criterion [16], where the N-enhanced resolution results in $\alpha =-\pi \left(1+\frac{1}{1000}\right)=-3.1447$ and ∆=−0.001π.

If the proposed Kth-order intensity product is applied to Figure 6a, as shown in Equation 6, then more resolvable frequencies are allowed, as discussed in Figure 5. For K = 100, the resolution of $I_{1000}$ for N = 1000 is 10 times enhanced to π/10,000, as shown by the blue and red dotted curves in Figure 5c. Thus, the unresolvable $f^{\prime \prime }$ positioned between $f_{0}$ and $f^{\prime }$ in Figure 5b is now resolvable, as shown by the green curve. This may sound awkward because we believe the measurement cannot retrospectively affect the optical system (interference). As the quantum eraser has been intensively studied over the last several decades for the mysterious phenomenon of the cause-effect relation [34,35,36], the enhanced resolution by the proposed intensity product method looks mysterious, too. Unlike the polarization-basis projection-based quantum erasers in ref. [36], however, the projection measurement in Figure 2, Figure 3, Figure 4, Figure 5 and Figure 6 is for the Gaussian-distributed statistical ensemble inside the MZI in Figure 1a, where the intensity product satisfying the SNL beats the Heisenberg limit in resolution, as shown in Figure 6c.

**Figure 7:**
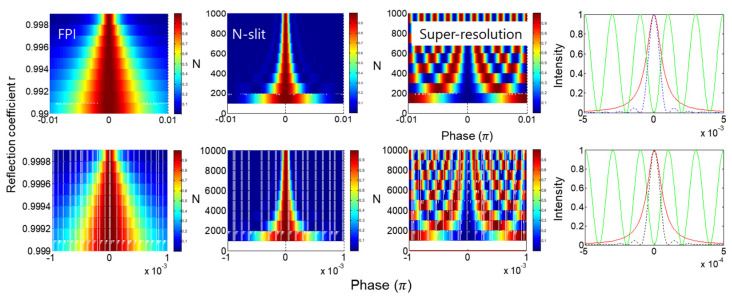
Comparison between FPI and N-slit interferometer. (**Left-end**) FPI. (**Middle-left**) N-slit interferometer. (**Middle-right**) super-resolution. (**Right-end**) fringes. Red: FPI; dotted: N-slit; green: super-resolution.

In Figure 7, FPI, N-slit interferometer, and super-resolution [12,13,14,25,37] are numerically investigated for the corresponding parameters of the resolution limit. The top (bottom) row is for less (more) dense cases with N. The left-end and middle-left columns are for FPI and N-slit cases [16]. The middle-right column is for the super-resolution of quantum sensing [37]. The right-end column is for comparison between them. For FPI, the transmitted intensity is $I_{T}\left(r\right)=1/\left[1+{\left(2r/(1-r^{2})\right)}^{2}{sin}^{2}\delta \right]$, where $\delta =2k_{d}d$ is the phase gain between cavity mirrors separated by distance d, and r is the reflection coefficient of the cavity mirror. For super-resolution [37], a typical MZI is reconfigured for the quantum eraser with orthogonal polarization bases [35,36], where the MZI output is divided into K folds for polarization-basis projection measurement through a polarizer [35,36,37]; otherwise, a single-photon-resolving detector must be used [14,25].

In the right-end column of Figure 7, the resolution of FPI (N slit) is represented by the red (blue-dotted) curve, representing the same FWHM. Thus, N = 1000 (10,000) corresponds to r = 0.999 (0.9999) in FPI, satisfying the same relation between N and r. Practically, however, FPI has a higher-order benefit with longer d, surpassing the grating-based spectrometer in achievable resolution. The N number in the middle-right column represents the number of divided fields for the intensity product. This intensity product is the same as SNL in Figure 5 and Figure 6, but the phase control of each divided field is required [37], resulting in N00N-based photonic de Broglie waves [20,21,22,23,24]. In the right-end column, super-resolution is represented by the green curve, demonstrating the same resolution as the N-slit system and FPI. Unlike the projection measurement in super-resolution and SNL, the FPI and N-slit interferometers are for amplitude superposition-based first-order (K = 1) intensity correlation, as shown in Equation (5) for Figure 4 [16]. As a result, either N wave superposition for the first-order intensity correlation or N projection measurement-based super-resolution shows the same Heisenberg limit in resolution. This fact has never been discussed yet.

## 5. Discussion

Unlike super-resolution [37], the proposed intensity-product-based SNL completely excludes phase relations between fields and thus satisfies the statistical ensemble of measurement events. As demonstrated in an experiment by Hanbury-Brown and Twiss [38], the advantage of the proposed intensity product method over the amplitude interference in FPI is the phase variation independence among fields due to the phase-independent identical intensities in Born’s rule [33]. This benefit has already been applied to optical spectrometers [37] and to quantum technologies using entangled photon pairs, even though the phase relation between entangled photons is still unknown. Thus, the SNL applied to the N-slit interference fringes in Figure 6c surpasses the maximum resolution achievable by quantum sensors confined by the Heisenberg limit. This unprecedented resolution can, of course, be applied to conventional grating-based spectrometers or FPI-based wavelength meters.

The technical advantage of the proposed intensity-product-based sensing method can also be found in a Si-photonics-integrated optical chip [39]. In general, the resolution of FPI strongly depends on the reflection coefficient of the cavity mirror, as shown in Figure 7. The discrete N fields from an N-slit (grating) system are an extreme case of FPI due to the same amplitudes, resulting in the π/N resolution (see the last column in Figure 7), which is equal to the Heisenberg limit in quantum sensing [14]. However, FPI and the N-slit system are optically bulky and extremely sensitive to the phase variation caused by environments, i.e., temperatures, mechanical vibrations, and air turbulences. Thus, Si-photonics can replace the bulky spectrometer for a robust micro-sensor beyond the diffraction limit. Moreover, the proposed sensing technique can also be applied to remote sensors such as a Doppler Lidar and hazardous gas detector to extend operational distance.

**Figure 8:**
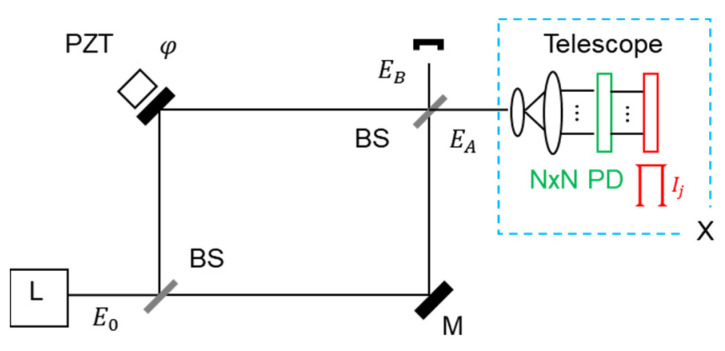
Schematic of intensity product measurements for a potential application. PD: 2D arrayed photodiode composed of N × N pixels.

For potential applications of the proposed intensity-product-based SNL, block ‘X’ in Figure 1a can be replaced by a two-dimensional photo sensor such as a conventional image sensor in a CCD camera, as shown in Figure 8. For this, an electronic circuit must be followed by a 2D photodiode for the intensity product of all sensor pixel data. As shown in ref. [29], no difference exists between the single photon and CW lights in fringe visibility. This means that the N × N division-caused intensity reduction in each channel does not ruin the original fringe visibility. A resolution enhancement factor η is the same as the square root of the pixel number of the sensor, resulting in η=1000 for a 1000 × 1000 off-the-shelf image sensor. The other application of the proposed method can be found in optical quantum communications for dense coding based on phase manipulations of coherent photons via a noiseless linear amplifier [40], where the noise figure can be enhanced by the proposed intensity-product measurements. For compatibility with Figure 1, an MZI-based secured optical key distribution protocol using a double unitary transformation may also be a good candidate for a round-trip configuration of MZI [41].

## 6. Conclusions

An intensity-product-based optical sensing method was proposed to surpass the limited resolution in conventional spectrometers. For the projection measurements of interference fringes, the SNL was satisfied not only for an MZI but also for an N-slit interferometer. The N-slit interference was analyzed for the same resolution as super-resolution in quantum sensing satisfying the Heisenberg limit. Due to the same physics of discrete phase relation between N waves, FPI was also compared with the N-slit interferometer, resulting in an equivalent parameter relation between reflection coefficient ‘r’ and slit number ‘N.’ Finally, the intensity product applied to the conventional spectrometer was numerically demonstrated for beating quantum sensors in terms of resolution. Due to the satisfaction of the statistical ensemble by the proposed intensity product sensing method, a simple electronic circuit for the Kth-order intensity product of an interference fringe from the spectrometer might be applied for the $\sqrt{K}$-enhanced resolution beyond the Heisenberg limit. Due to the coherence feature, a cw frequency modulation in Radar technology might be useful for the proposed intensity-product method to extend operational distance.

## Data Availability

All data generated or analyzed during this study are included in this published article.
